# Knockdown of kinesin family member 4A inhibits cell proliferation, migration, and invasion while promoting apoptosis of urothelial bladder carcinoma cells

**DOI:** 10.1002/cam4.5932

**Published:** 2023-04-11

**Authors:** Chen Zhang, Maoyu Wang, Yidie Ying, Fang Meng, Hongliang Gao, Shuxiong Zeng, Yasheng Zhu, Anwei Liu, Zhensheng Zhang, Chuanliang Xu

**Affiliations:** ^1^ Department of Urology, Changhai Hospital Naval Military Medical University Shanghai 200433 China; ^2^ Department of Critical Care Medicine Hospital of Southern Theatre Command of PLA Guangzhou 510010 China

**Keywords:** AKT serine/threonine kinase, kinesin family member 4A, urothelial bladder carcinoma, Yes1 associated transcriptional regulator

## Abstract

**Background:**

Kinesin family member 4A (KIF4A) is upregulated in a variety of cancers. However, its expression and potential downstream targets in urothelial bladder carcinoma (UBC) remain unclear.

**Methods:**

Expression data of KIF4A in UBC and noncancerous tissues were downloaded from the GEPIA database. Cell proliferation, migration, invasion, and apoptosis of T24 and 5637 UBC cells were examined using wound healing, transwell, colony formation, CCK‐8, and flow cytometry assays. KIF4A and potential downstream genes were analyzed using qRT‐PCR, western blot analysis, and immunohistochemistry.

**Results:**

In UBC samples, KIF4A expression was significantly higher than in corresponding noncancerous samples. UBC patients with high KIF4A expression had poor cancer‐specific survival and overall survival. Knockdown of KIF4A significantly inhibited proliferation and promoted apoptosis of UBC cells, accompanied by dephosphorylation of AKT and increased the protein level of proapoptotic factors. Additionally, knockdown of KIF4A reduced migration and invasion of UBC cells whereas overexpression of KIF4A exhibited opposite effects, along with altered protein level in epithelial‐mesenchymal transition‐related genes. Furthermore, overexpression of YAP1 promoted KIF4A expression whereas knockdown of YAP1 suppressed KIF4A expression in UBC cells. Alternatively, KIF4A knockdown reduced YAP1 nuclear protein level whereas KIF4A overexpression suppressed YAP1 phosphorylation and facilitated YAP1 nuclear translocation.

**Conclusions:**

KIF4A upregulation correlates with poor prognosis of UBC. Knockdown of KIF4A inhibits proliferation, migration, and invasion of UBC cells while inducing apoptosis possibly through dephosphorylation of AKT, changes in EMT‐related genes, and interaction with YAP1.

## 1 INTRODUCTION

Urothelial bladder carcinoma (UBC) is the most common neoplasm of the urinary system.^1^ Approximately 5%–10% of UBC cases present metastases at the time of diagnosis. After radical cystectomy, about 50% of patients experience local or distant tumor relapse.^2^ Despite the development of therapeutic approaches, nearly 50%–70% of cases relapse within 5 years of recovery.^3^ Therefore, it is important to identify new therapeutic targets for UBC.

Kinesin family member 4A (KIF4A) is located on chromosome Xq13.1, encoding a protein containing 1232 amino acids. KIF4A participates in multiple cellular processes, such as centrosome assembly and spindle formation in mitosis, chromosomal separation and concentration, as well as DNA damage repair.^4^ KIF4A dysregulation occurs in various tumors, including lung,^5^ liver,^6^ breast,^7^ and bladder cancer.^8^ Yes1 associated transcriptional regulator (YAP1) is an important component of the Hippo signaling pathway that is highly associated with cell growth. YAP1 induces KIF4A transcription by binding to the KIF4A promoter via TEA domain transcription factor 4 (TEAD4), contributing to the progression and poor prognosis of esophageal squamous cell carcinoma.^9^ However, the role of KIF4A in UBC metastasis and the underlying mechanism of KIF4A dysregulation in UBC remain largely unknown.

Abnormal or incomplete mitosis may induce cell apoptosis. KIF4A silencing may lead to apoptosis by inducing spindle defects, dysfunction in prometaphase organization, metaphase chromosomal misalignment, and chromosomal mis‐segregation.^10^ A recent study has shown that KIF4A silencing reduces p‐AKT level in gastric cancer.^11^ Since AKT regulates cell proliferation and apoptosis by phosphorylating multiple downstream targets, such as P21, Bax, and Bcl‐2,^12^ we hypothesized that KIF4A might affect the apoptosis of UBC cells through AKT.

In this study, we investigated the expression of KIF4A in human UBC and the role of KIF4A in UBC cell proliferation, migration, invasion, and apoptosis. To identify the potential mechanisms underlying the role of KIF4A in UBC development, we examined the alterations in the protein level of apoptosis and epithelial‐mesenchymal transition (EMT)‐related genes as well as the interaction between KIF4A and YAP1. Our results suggest that KIF4A contributes to UBC development and metastasis possibly through the activation of AKT and interaction with YAP1.

## 2 MATERIALS AND METHODS

### 2.1 Specimens and data collection

KIF4A expression data of 404 UBC tumor samples and 28 noncancerous tissue samples were acquired from the (GEPIA) Gene Expression Profiling Interactive Analysis database. A total of 26 UBC patients who underwent radical cystectomy between June 2013 and May 2021 at the department of urology at The First Affiliated Hospital of Naval Medical University (Shanghai, China) were recruited to validate differential expression of KIF4A between bladder cancer and noncancerous tissue samples. RNA sequencing was used to validate the differential expression of KIF4A in 10 patients admitted between June 2013 and May 2014 (Table S1), and western blot analysis was used to validate the differential expression in 16 patients admitted between May 2020 and May 2021 (Table S2). A total of 232 bladder cancer and adjacent noncancerous specimens were obtained from our hospital for immunohistochemistry (IHC) analysis (Table 1). A tissue microarray was prepared. The specimens were collected during transurethral resection of bladder tumors or radical cystectomy from 1998 to 2018, including 148 muscle‐invasive bladder cancer, 84 non‐muscle‐invasive bladder cancer, 185 high‐grade bladder cancer, and 47 low‐grade bladder cancer.

**TABLE 1:** Association of KIF4A expression with clinicopathological characteristics of patients with urothelial bladder carcinoma.

Variables	KIF4A expression		
	Low (*n* = 126)	High (*n* = 106)	*p‐*value (χ^2^ test)
Gender			
Male	113 (54.07%)	96 (45.93%)	0.822
Female	13 (56.52%)	10 (43.48%)	
Age, years			
≤65	27 (57.45%)	20 (42.55%)	0.743
>65	99 (53.51%)	86 (46.49%)	
Tumor size			
<3 cm	48 (64.00%)	27 (36.00%)	0.048
≥3 cm	78 (49.68%)	79 (50.32%)	
Tumor stage			
<T2	61 (72.62%)	23 (27.38%)	<0.001
T2–T4	65 (43.92%)	83 (56.08%)	
Tumor grade			
Low	32 (68.09%)	15 (31.91%)	0.048
High	94 (50.81%)	91 (49.19%)	
Recurrence history			
No	92 (57.86%)	67 (42.14%)	0.120
Yes	34 (46.58%)	39 (53.42%)	

### 2.2 Cell lines and cell culture

UBC cell lines were obtained from American Type Culture Collection (Manassas) and cultured in DMEM (T24 and HT‐1376 cells), RPMI‐1640 (5637 cells), MEM (J82 and UM‐UC‐3 cells), or L15 medium (BIU‐87 cells) supplemented with 10% of fetal bovine serum (FBS; Gibco; Thermo Fisher Scientific) in an atmosphere of 5% CO_2_ at 37°C.

### 2.3 Preparation of nuclear and cytoplasmic extract

Nuclear and cytoplasmic extracts were prepared using NE‐PER™ nuclear and cytoplasmic extraction kit (Thermo Scientific). Pre‐chilled 200 μL CER1 reagent was added to the cells, followed by vortex shaking at high speed for 15 s. After incubation on ice for 10 min, 11 μL of CERII reagent was added to the cells, vortexed for 5 s, then incubated on ice for 1 min. After centrifuging at 16,000 rpm for 5 min, the supernatant was transferred to a fresh pre‐cooled centrifuge tube to store on ice or at −80°C for further experiments. 100 μL of pre‐chilled NER was Add to the precipitate, resuspended with high speed vortex for 15 s, incubated on ice for 10 min, and vortexed again at high speed for 15 s. This process was repeated four times. The nucleoplasmic protein extract was obtained by centrifuging the solution at 16,000 rpm for 10 min and keep on ice or stored at −80°C for subsequent experiments.

### 2.4 Quantitative reverse transcription PCR (qRT‐PCR)

Total RNA was isolated from UBC cells using TRIzol reagent (Invitrogen), followed by cDNA synthesis using an all‐in‐one RT super‐mix perfect kit (Vazyme) according to the manufacturer's protocol. PCR was performed using a Taq Pro universal SYBR qPCR master mix (Vazyme) on Applied Biosystems step one plus (Agilent Technologies). Relative gene expression was determined using the 2^−ΔΔCT^ method. The primer sequences were as follows: KIF4A 5′‐TGGTGTGGAAACAAGCAGTGTG‐3′ (forward), 5′‐GGAATCCTGGGTCCGTTCA‐3′ (reverse); ITGB1, 5′‐CCTACTTCTGCACGATGTGATG‐3′ (forward), 5′‐CCTTTGCTACGGTTGGTTACATT‐3′ (reverse); GAPDH, 5′‐ACCACAGTCCATGCCATCAC‐3′ (forward), 5′‐TCCACCACCCTGTTGCTGTA‐3′ (reverse); β‐actin, 5′‐CATGTACGTTGCTATCCAGGC‐3′ (forward), 5′‐CTCCTTAATGTCACGCACGAT‐3′ (reverse).

### 2.5 IHC

IHC was performed to detect KIF4A expression in the tissue microarray containing 232 UBC tumor tissue samples. The microarray was incubated with anti‐KIF4A primary antibody (1:400; ab122227, Abcam) and stained using an IHC kit (BioGenex) following the manufacturer's protocol. The IHC scores of the cytoplasm and nuclei were calculated, respectively, as 0 × % non‐stained + 1 × % weakly stained + 2 × % moderately stained + 3 × % strongly stained, ranging from 0 to 300.^13^ Patients were divided into high and low KIF4A expression groups according to the median (240) of the sum of cytoplasmic IHC score and nuclear IHC score.^13^

### 2.6 Gene knockdown and overexpression

Small interference RNAs (siRNAs) against KIF4A or YAP1 were purchased from GenePharma. The siRNA sequences were as follows: KIF4A: siRNA‐1: sense, 5′‐CUGCAGAGCAAGAGAAUGATT‐3′, antisense, 5′‐UCAUUCUCUUGCUCUGCAGUA‐3′; siRNA‐2: sense, 5′‐CAGCAAAGAAGGAUGCCAATT‐3′, antisense, 5′‐UUGGCAUCCUUCUUUGCUGUC‐3′; siRNA‐3: sense, 5′‐GCUGGUUGAGUUGAAUAAATT‐3′, antisense, 5′‐UUUAUUCAACUCAACCAGCUC‐3′; nonspecific siRNA: sense, 5′‐UUCUCCGAACGUGUCACGUTT‐3′, antisense, 5′‐ACGUGACACGUUCGGAGAATT‐3′; YAP1 siRNA: sense, 5′‐CAGGUGAUACUAUCAACCATT‐3′, antisense, 5′‐UGGUUGAUAGUAUCACCUGTT‐3′. Lentiviral vectors expressing KIF4A (#31640‐1) or YAP1 (#14517‐2) were purchased from Genechem. UBC cells were transfected with siRNAs or lentiviral vectors using RNAiMAX or lipofectamine (Invitrogen) following the manufacturer's instructions.

### 2.7 Western blot analysis

Protein concentration was measured using the BCA method. Equal amount of each protein sample was separated on SDS‐PAGE and transferred onto PVDF membranes (Bio‐Rad). The membranes were washed five times with TBST, 5 min each, followed by blocking with 5% skimmed milk in TBST for 1 h at 37°C. Then, the membranes were incubated with primary antibody against KIF4A (1:2500, ab124903), GAPDH (1:2500, ab9485), β‐actin (1:50000, AC026, Abclonal), ITGB1 (1:4000, 12,594‐1‐AP, Proteintech), YAP1 (1:5000, ab52771), p‐YAP (1:2000, S127, Cell Signaling Technology (Cell Signaling Technology)), AKT (1:2000, 2920, Cell Signaling Technology), p‐AKT (Ser473 1:2000, 4060, Cell Signaling Technology), cleaved‐caspase 3 (1:2000, 19,677‐I‐AP, Proteintech), Bax (1:5000, 50,599‐2‐Ig, Proteintech), Bcl‐2 (1:5000, 60,178‐1‐Ig, Proteintech), PARP (1:2000, 46D11, Cell Signaling Technology), cleaved‐PARP (1:1000, Asp214, Cell Signaling Technology), P21 (1:1000, GB11153, Servicebio), E‐cadherin (1:1000, ab76055), N‐cadherin (1:2000, GB12135, Servicebio), or vimentin (1:1000, GB11192, Servicebio) overnight at 4°C. After TBST washes, the membranes were incubated with HRP‐conjugated anti‐rabbit or anti‐mouse secondary antibody (1:3000, Cell Signaling Technology) for 2 h at 4°C. The protein bands were visualized using an enhanced chemiluminescence detection kit (Millipore) and analyzed using the ChemiDoc MP system (Bio‐Rad). The relative levels of the proteins were quantified by normalizing the densitometry to controls using ImageJ 1.53.

### 2.8 Cell counting kit‐8 (CCK‐8) assay

Cell proliferation was assessed using CCK‐8 (Dojindo) following the manufacturer's instructions. Cells were seeded in 96‐well plates at a density of 3 × 10^3^ cells/well. After treatment, cells were incubated with 20  μL CCK‐8 reagent for 3 h. The absorbance was measured at a wavelength of 495 nm using an EnSpire microplate reader (PerkinElmer).

### 2.9 Wound healing assay

UBC cells were seeded in 6‐well plates. When the cells achieved 80% confluency, a scratch was made using a 200‐μL pipette tip. Cells were then cultured in FBS‐free medium. Images were acquired at 0, 24, 48, or 72  h after scratching.

### 2.10 Transwell assay

Cell invasion was assessed using the Matrigel‐coated transwell chamber (Millipore). Briefly, 4 × 10^4^ cells were seeded in the upper chamber containing 500  μL FBS‐free medium. A total of 1  mL of complete medium was added into the lower chamber.

Cell migration was assessed using the transwell chamber (Corning). 3 × 10^4^ cells were seeded in the upper chamber containing 500  μL FBS‐free medium. A total of 1  mL of complete medium was added into the lower chamber. T24 and 5637 cells were allowed to invade and migrate for 18 and 48 h, respectively. Then, the cells remaining in the upper chamber were removed using a cotton swab. Cells adhering to the lower surface were fixed with 4% formalin for 20 min and then stained with 0.1% crystal violet for 30 min. Images were acquired in 10 randomly selected fields, and cells were counted.

### 2.11 Colony formation assay

Cells were seeded in 3.5‐cm plates at a density of 5 × 10^2^ cells/well. At 2 weeks after transfection, cells were fixed using 10% methanol, followed by staining with 0.1% crystal violet. Colony formation was analyzed using a microscope.

### 2.12 Flow cytometry assay

Cells were harvested at 72 h after KIF4A or control siRNA transfection. Cell apoptosis was examined using an Annexin V/FITC and PI apoptosis kit (Lianke Biotech). The results were analyzed using a MACSQuant analyzer and FlowJo v10.8 software.

### 2.13 Statistical analysis

Data were expressed as the mean ± standard error of the mean. Statistical analysis was carried out using Prism software (version 8.4.3). Comparisons of measurement data between two groups were conducted using Student's *t*‐test. The chi‐squared test was performed to analyze quantitative data and the association of clinicopathological parameters with KIF4A expression level. Overall survival (OS) and cancer‐specific survival (CSS) were analyzed using Kaplan–Meier analysis and log‐rank test. A *p* value < 0.05 (two‐sided) was considered statistically significant.

## 3 RESULTS

### 3.1 KIF4A is upregulated in UBC and associated with poor prognosis of UBC patients

**FIGURE 1:**
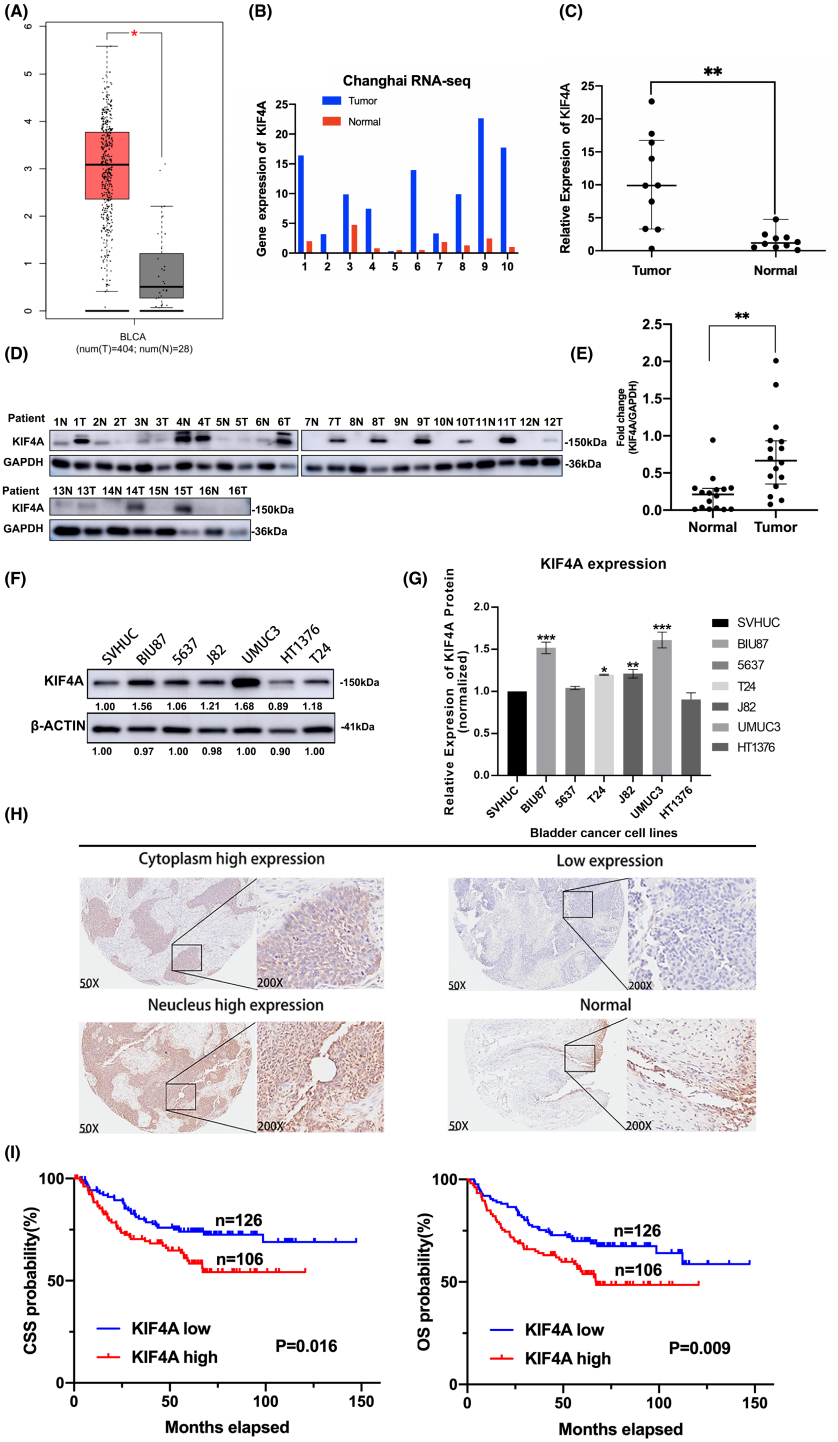
Kinesin family member 4A (KIF4A) was upregulated in urothelial bladder carcinoma (UBC) and associated with poor prognosis of UBC patients. (A) Comparison of KIF4A mRNA expression between bladder cancer samples (*n* = 404) and normal urothelium samples (*n* = 28) from the GEPIA (Gene Expression Profiling Interactive Analysis database). T, tumor; N, normal. (B,C) Comparison of KIF4A mRNA expression between bladder cancer samples and normal urothelial samples from 10 UBC patients by RNA sequencing. (D,E) Western blot analysis was conducted to determine KIF4A protein expression in 16 pairs of bladder cancer samples and noncancerous samples. T, tumor; N, noncancerous. GAPDH was used as an internal reference. (F,G) Western blot analysis was conducted to measure KIF4A protein expression in normal urothelial cell line SVHUC, non‐muscle‐invasive bladder cancer cell line BIU‐87, and five different muscle‐invasive bladder cancer cell lines (5637, J82, UMUC3, HT1376, and T24). β‐Actin was used as an internal reference. Data are expressed as the mean ± standard error of the mean (SEM). *p < 0.05, **p < 0.01, ***p < 0.001; *n* = 3. (H) Immunohistochemical staining was performed to determine KIF4A expression in human UBC samples and normal urothelial samples. Representative images are shown. UBC patients were categorized into low (IHC score ≤ 240, *n* = 126) and high (IHC score > 240, *n* = 106) KIF4A expression groups. (I) Survival analysis was conducted to examine the correlation of KIF4A expression with cancer‐specific survival and overall survival of patients. CSS, cancer‐specific survival; OS, overall survival.

To explore the involvement of KIF4A in UBC, we compared KIF4A mRNA expression between bladder cancer tissue and normal bladder tissue from the GEPIA. We found that KIF4A mRNA expression was remarkably upregulated in bladder cancer tissue compared with that in normal bladder tissue (Figure 1A). This was confirmed in the samples of 10 UBC patients from our hospital (Figure 1B,C). Consistently, in 16 UBC patients at our hospital, KIF4A protein was generally highly expressed in tumor tissue but barely detectable in paired normal bladder tissue (Figure 1D,E). In addition, KIF4A protein was extensively expressed in UBC cell lines, including BIU87, 5637, J82, UMUC3, HT1376, and T24, as well as in normal urothelial cells SVHUC. Specifically, KIF4A protein level in BIU87, T24, J82, and UMUC3 cells were markedly increased compared with that in SVHUC cells (*p* < 0.05; Figure 1F,G). Furthermore, IHC staining showed that KIF4A protein was expressed in both nuclear and cytoplasm of UBC cells (Figure 1H). Based on the mean IHC score, UBC patients were categorized into low (IHC score ≤ 240, *n* = 126) and high (IHC score > 240, *n* = 106) KIF4A expression groups. We found that high KIF4A expression was significantly associated with tumor size >3 cm (*p* = 0.048) and advanced tumor grade (*p* = 0.048) and tumor stage (*p* < 0.001) (Table S1). Survival analysis showed that high KIF4A expression was significantly correlated to poor CSS (*p* = 0.016) and OS (*p* = 0.009) **(**Figure 1I **)**. These results suggest that KIF4A upregulation is associated with disease severity and poor prognosis of patients with UBC.

### 3.2 Knockdown of KIF4A inhibits proliferation while promoting apoptosis of UBC cells

**FIGURE 2:**
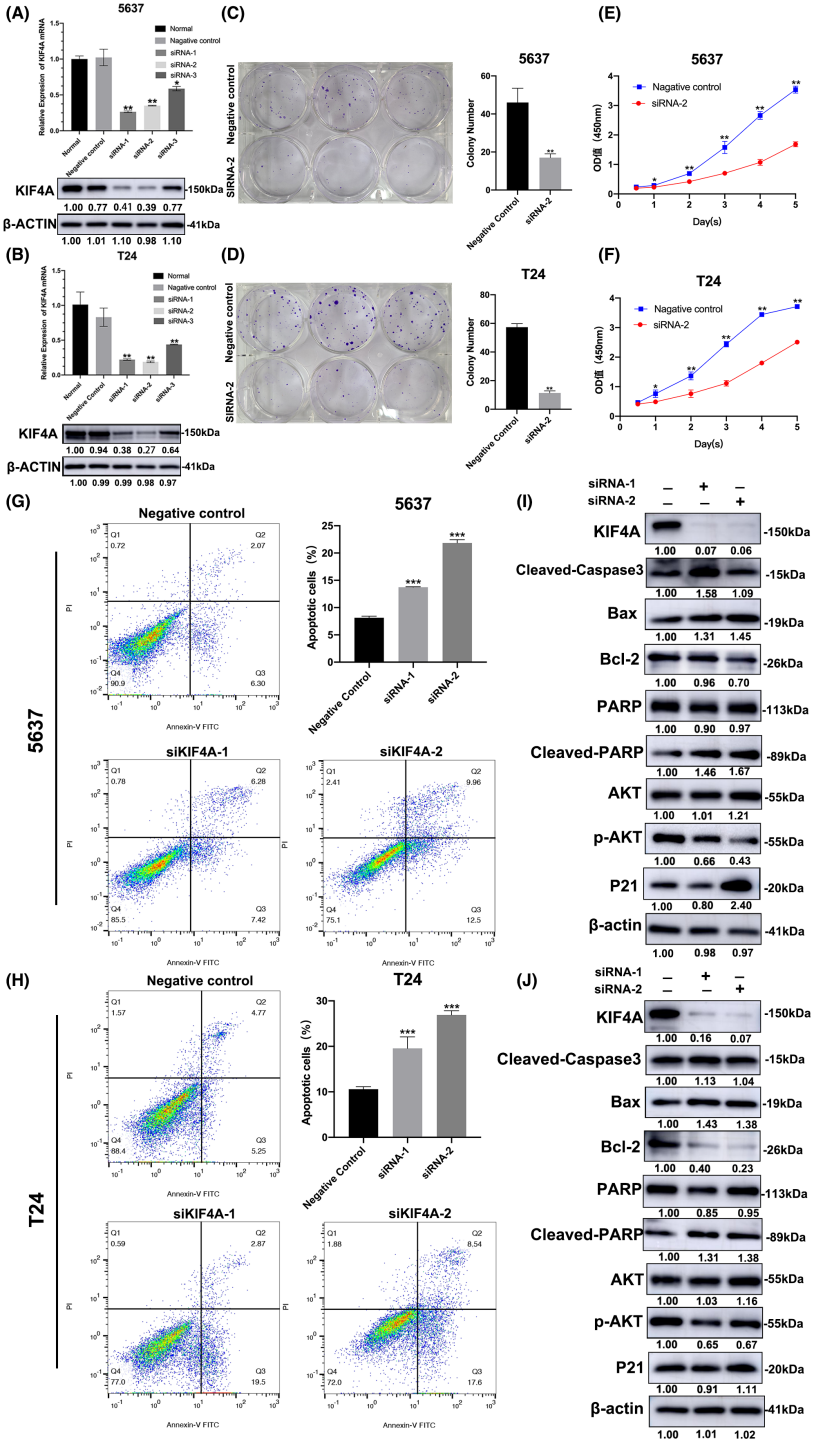
Knockdown of KIF4A inhibited colony formation and proliferation while promoting apoptosis of UBC cells. (A,B) 5637 (A) and T24 (B) cells were transfected with siRNA‐1, siRNA‐2, or siRNA‐3 against KIF4A. Western blot analysis was conducted to measure KIF4A protein level in the cells. (C–F) 5637 (C,E) and T24 (D,F) cells were transfected with siRNA‐2 against KIF4A. Colony formation assay (C,D) and CCK‐8 assay (E,F) were performed to examine cell proliferation. (G,H) 5637 (G) and T24 (H) cells were transfected with siRNA‐1 or siRNA‐2 against KIF4A. Flow cytometry analysis was carried out to examine cell apoptosis. (I,J) Western blot analysis was conducted to determine protein expression of p‐AKT (Ser473), total AKT, Bcl‐2, Bax, cleaved‐PARP, PARP, cleaved‐caspase‐3, YAP1, p‐YAP1, and P21 in 5637 (I) and T24 (J) cells. β‐Actin was used as an internal reference. Data are expressed as the mean ± SEM. *p < 0.05, **p < 0.01, ***p < 0.001 versus negative control; *n* = 3.

To explore the role of KIF4A in UBC, we silenced KIF4A expression in 5637 **(**Figure 2A **)** and T24 **(**Figure 2B **)** cells, respectively, and found that siRNA‐2 exhibited the most significant knockdown efficiency among three siRNAs. Colony formation assay and CCK‐8 assay showed that knockdown of KIF4A considerably inhibited cell proliferation of both cell lines **(**Figure 2C–F **)**. Meanwhile, knockdown of KIF4A markedly enhanced cell apoptosis of both UBC cell lines **(**Figure 2G,H **)**. Of note, siRNA‐2 outperformed siRNA‐1 in promoting apoptosis of UBC cells. These data suggest that KIF4A is required for the survival of UBC cells.

### 3.3 Knockdown of KIF4A deactivates AKT signaling in UBC cells

Because KIF4A silencing reduces p‐AKT level in gastric cancer,^11^ we investigated the effect of KIF4A silencing on AKT activation and the protein level of downstream apoptosis‐related genes in UBC cells. Western blot analysis showed that knockdown of KIF4A substantially attenuated AKT phosphorylation in both UBC cell lines, with the total AKT protein level remaining unchanged. In the meantime, knockdown of KIF4A significantly enhanced the protein level of proapoptotic cleaved caspase 3, Bax, cleaved PARP, and p21 while suppressing protein level of cell proliferation‐related Bcl‐2 and p‐YAP1 in both cell lines (Figure 2I,J). Again, siRNA‐2 was superior to siRNA‐1 in AKT dephosphorylation.

### 3.4 KIF4A promotes cell migration and invasion of UBC cells

**FIGURE 3:**
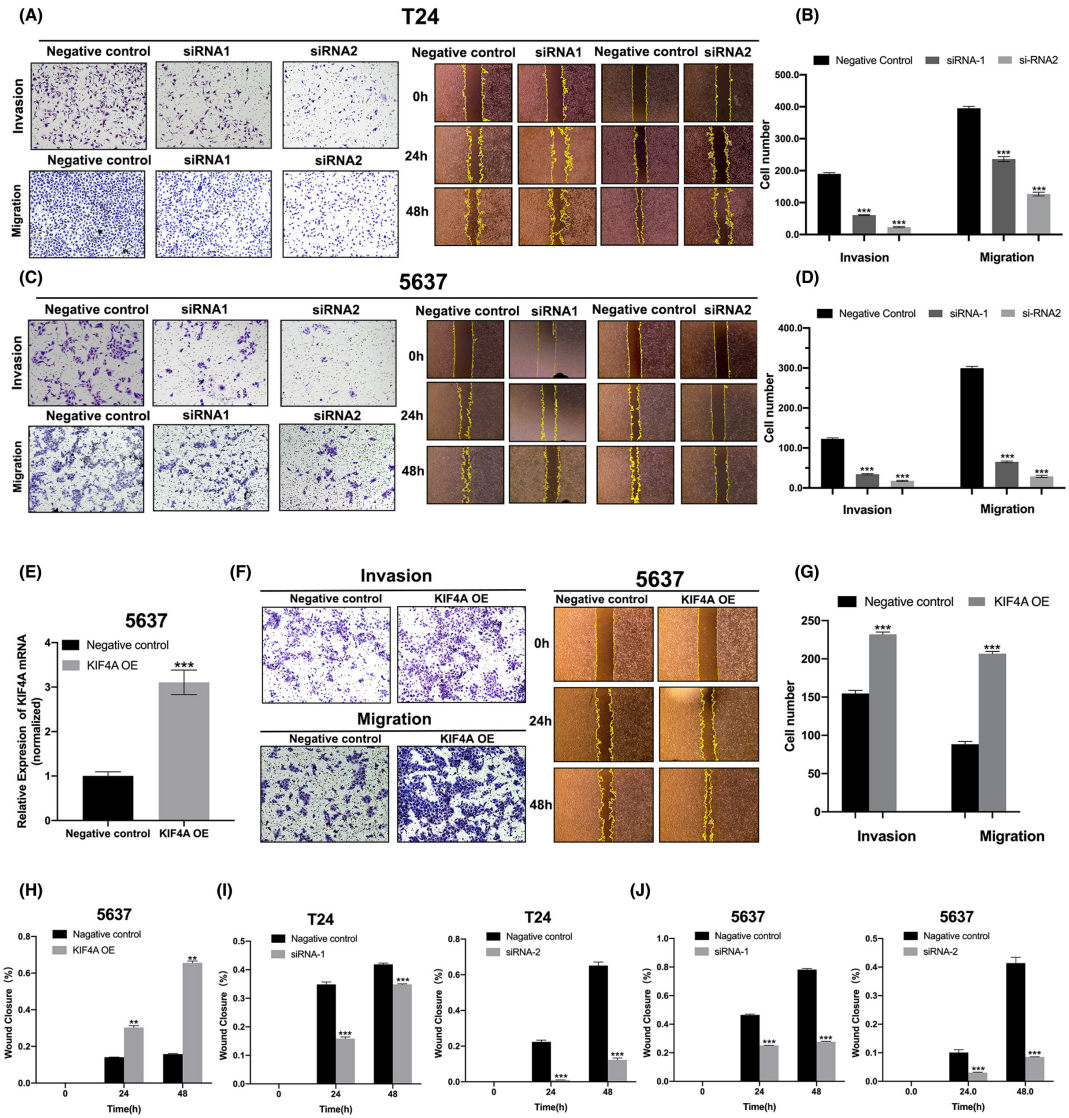
KIF4A promoted UBC cell migration and invasion. (A–D) T24 (A,B) and 5637 (C,D) cells were transfected with siRNA‐1 or siRNA‐2 against KIF4A. Transwell assay and wound healing assay were performed to examine cell invasion and migration. Representative images are shown. Magnification × 100. (E) 5637 cells were transfected with lentiviral vectors expressing KIF4A. (F) Transwell assay and wound healing assay were performed to examine cell invasion and migration. (G,H) Quantification of F. (I,J) T24 (I) and 5637 (J) were transfected with siRNA‐2 against KIF4A. A wound healing assay was performed to examine cell migration. Data are expressed as the mean ± SEM. **p < 0.01, ***p < 0.001 versus negative control; *n* = 3. OE, overexpression.

Then, we explored the role of KIF4A in UBC cell migration and invasion. Transwell and wound healing assays showed that KIF4A silencing markedly reduced cell invasion and migration of both T24 and 5637 cells (Figure 3A–D). On the contrary, KIF4A overexpression (Figure 3E) substantially promoted cell invasion and migration of 5637 cells (Figure 3E–H). Time‐dependent trends were observed in the promoting effect of KIF4A overexpression on migration of 5637 cells (Figure 3H) and the inhibitory effect of KIF4A silencing on migration of both T24 and 5637 cells (Figure 3I,J).

### 3.5 Knockdown of KIF4A modulates EMT‐related genes in UBC cells

**FIGURE 4:**
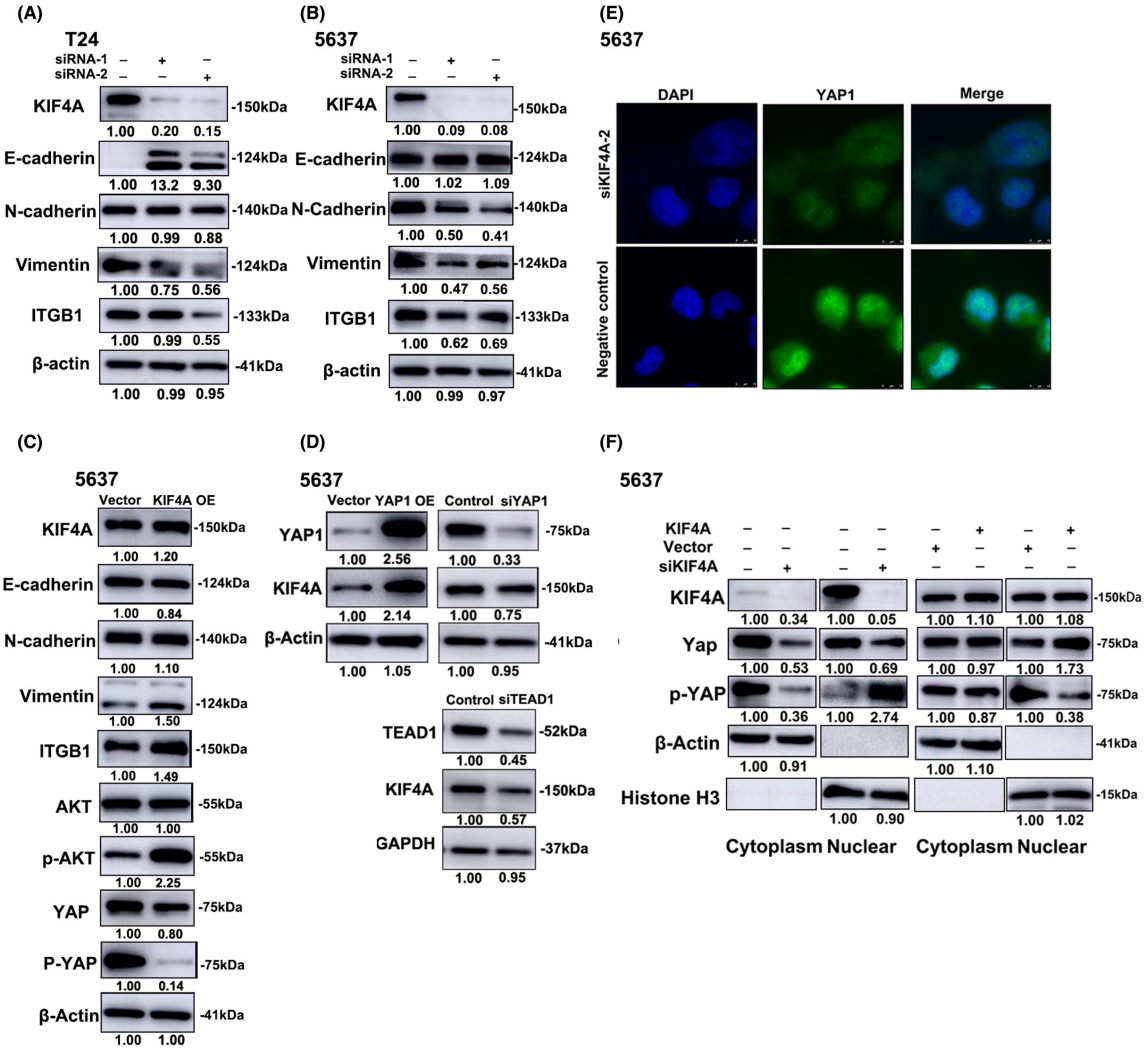
KIF4A regulated the protein level of EMT‐related genes and interacted with Yes1 associated transcriptional regulator 1 (YAP1). (A,B) T24 (A) and 5637 (B) cells were transfected with siRNA‐1 or siRNA‐2 against KIF4A. Western blot analysis was performed to determine the protein level of KIF4A, E‐cadherin, N‐cadherin, vimentin, ITGB1, and β‐actin. (C) 5637 cells were transfected with lentiviral vectors expressing KIF4A. Western blot analysis was conducted to determine the protein level of KIF4A, E‐cadherin, N‐cadherin, vimentin, ITGB1, AKT, p‐AKT, YAP1, p‐YAP1, and β‐actin. (D) 5637 cells were transfected with lentiviral vectors expressing YAP1 or siRNA against YAP1 (siYAP1). Western blot analysis was conducted to determine the protein level of YAP1, KIF4A, TEAD1, β‐actin, and GAPDH. (E) 5637 cells were transfected with siRNA against KIF4A. Immunofluorescence staining was performed to detect YAP1 protein level. (F) 5637 cells were transfected with lentiviral vectors expressing KIF4A or siRNA against KIF4A as indicated. Western blot analysis was carried out to determine the cytoplasmic and nuclear protein level of KIF4A, YAP1, and p‐YAP1. β‐actin and Histone H3 were used as cytoplasmic and nuclear internal references, respectively.

Increased ability of migration and invasion are linked to EMT that is a key step in cancer metastasis.^14^ Thus, we sought to investigate whether KIF4A regulates the protein level of EMT‐related genes. We found that knockdown of KIF4A significantly suppressed protein level of N‐cadherin, vimentin, and integrin subunit beta 1 (ITGB1) while enhancing protein level of E‐cadherin in UBC cells (Figure 4A,B). By contrast, overexpression of KIF4A significantly boosted protein level of N‐cadherin, vimentin, and ITGB1 while diminishing protein level of E‐cadherin in UBC cells. In the meantime, overexpression of KIF4A dramatically increased AKT phosphorylation while reducing YAP1 phosphorylation (Figure 4C). These data suggest that KIF4A can potentially facilitate UBC metastasis by promoting EMT of UBC cells.

### 3.6 KIF4A interacts with YAP1 in UBC

Considering the regulatory role of YAP1/TEAD4 in KIF4A transcription in esophageal squamous cell carcinoma and the role of TEAD1 in chemoresistance of bladder cancer,^9^, ^15^ we sought to investigate whether YAP1/TEAD1 is involved in KIF4A dysregulation in UBC. We found that overexpression of YAP1 resulted in a 2.14‐fold increase in KIF4A protein level whereas knockdown of YAP1 or TEAD1 led to a < 50% reduction in KIF4A protein level in UBC cells **(**Figure 4D **)**. These data suggest that YAP1/TEAD1 are at least partially responsible for KIF4A upregulation in UBC. Cytoskeleton proteins can bind to phosphorylated YAP1 (p‐YAP1), inducing its cytoplasmic translocation and degradation and subsequent inhibition of cell proliferation.^16^ Our immunofluorescence data demonstrated that knockdown of KIF4A dramatically reduced YAP1 nuclear protein level in 5637 cells (Figure 4E), suggesting an interaction between KIF4A and YAP1 in UBC. Furthermore, western blot analysis showed that overexpression of KIF4A significantly suppressed YAP1 phosphorylation in both cytoplasm and nucleus of 5637 cells and remarkably increased YAP1 protein level in the nucleus. Opposite effects were observed in KIF4A‐silenced cells (Figure 4F). These data suggest that YAP1 is involved in KIF4A upregulation in UBC and that KIF4A contributes to UBC cell proliferation possibly by promoting YAP1 dephosphorylation and nuclear translocation.

## 4 DISCUSSION

This study explored KIF4A expression in human UBC and the role of KIF4A in UBC cell proliferation, migration, invasion, and apoptosis. We demonstrated that KIF4A protein was strongly expressed in human UBC tumor tissue and UBC cell lines compared with that in noncancerous tissue and normal urothelial cells, respectively. High KIF4A expression was significantly correlated with poor survival of patients with UBC. In vitro studies showed that knockdown of KIF4A hindered cell proliferation while promoting apoptosis of UBC cells. On the contrary, overexpression of KIF4A facilitated UBC cell migration and invasion. At the molecular level, knockdown of KIF4A reduced AKT phosphorylation and modulated EMT‐related proteins in UBC cells. Furthermore, KIF4A interacted with YAP1 in UBC cells. Taken together, our study suggests that KIF4A may promote UBC growth and metastasis, serving as a potential therapeutic target for UBC.

Apoptosis involves two major pathways, namely intrinsic and extrinsic pathway. Caspases‐3 is an important proapoptotic molecule and can cleave downstream targets such as PARP and P21.^17^ Bcl‐2 and Bax are the two factors that mediate the intrinsic apoptotic pathway. Bcl‐2 disassociated from Bax triggers the intrinsic apoptosis by modulating mitochondrial function.^18^ In this study, KIF4A silencing suppressed Bcl‐2 protein level while enhancing Bax protein level in UBC cells, accompanied with elevations in cleaved caspase‐3, cleaved PARP, and P21 protein level. Similarly, Zhang et al. have reported that knockdown of KIF4A inhibits tumor progression and promotes chemosensitivity of lung cancer cells by inducing P21 expression.^19^ Jin et al. have observed diminished Bcl‐2 protein level and increased Bax and cleaved caspase3 protein level in KIF4A‐silenced apoptotic ovarian cancer cells.^20^ Thus, targeting KIF4A is a promising therapeutic approach to cancer therapy by inducing apoptosis of tumor cells.

The transcription coactivator YAP1 is an important molecule in the Hippo pathway that regulates cell proliferation. In esophageal squamous cell carcinoma, KIF4A inhibits YAP1 phosphorylation, leading to enhanced nuclear YAP1 protein level.^21^ Consistently, we found that overexpression of KIF4A suppressed YAP1 phosphorylation and promoted YAP1 nuclear translocation in 5637 cells. In addition, YAP1 can modulate the transcription of anti‐apoptotic genes, such as the Bcl‐2 family member Bcl‐xL.^22^ We speculated that the Bcl‐2 downregulation in response to KIF4A silencing might be related to the reduction in YAP1 protein level. These observations suggest that KIF4A silencing promotes UBC cell apoptosis possibly by regulating the phosphorylation and cytoplasmic/nuclear translocation of YAP1.

AKT activation suppresses cytochrome c production through direct phosphorylation of Bax and subsequent suppression of mitochondrial membrane translocation of Bax.^23^ A recent study has shown that KIF4A silencing suppresses AKT activation in hepatocellular carcinoma.^24^ Therefore, we hypothesized that the AKT pathway might be involved in KIF4A silencing‐induced Bax upregulation in UBC cells. As expected, we found that KIF4A silencing inhibited AKT phosphorylation in UBC cells, along with Bax upregulation. Thus, KIF4A possibly prevents cell apoptosis through AKT activation, which needs to be verified by AKT inhibition in future studies.

EMT is associated with increased ability of cell migration and invasion, playing a key role in cancer metastasis.^25^ Acquisition of EMT phenotypes is related to the activation of the PI3K/AKT/mTOR pathway.^26^ However, the association of KIF4A with EMT in UBC remains unclear. In this study, overexpression or knockdown of KIF4A altered the protein level of EMT markers, suggesting that KIF4A could trigger EMT in UBC. In addition, KIF4A can transport ITGB1 in developing axons of cortical neurons.^27^ Our results showed that knockdown of KIF4A suppressed protein level of ITGB1, whereas overexpression of KIF4A enhanced protein level of ITGB1 in UBC cells. ITGB1 can activate the AKT/FAK/YAP pathway during cell apoptosis and facilitate EMT.^28^, ^29^ PI3K/AKT has been shown to regulate tumor cell invasion and metastasis by promoting ITGB3 expression.^30^ These findings, including ours, implies the involvement of ITGB1 in the role of KIF4A in apoptosis, migration, and invasion of UBC cells and further highlights the involvement of AKT and YAP1. However, these data need to be verified by loss of function analyses. In vivo studies are also required to assess the therapeutic potential of targeting KIF4A in UBC.

In conclusion, to the best of our knowledge, this study furtherly reported underlying mechanism that KIF4A facilitates cell proliferation, migration, and invasion of UBC cells and regulates the associated protein level of EMT, proliferation, and apoptosis‐related proteins, such as p‐AKT, ITGB1, p‐YAP, and YAP1. These results suggest that KIF4A is a promising therapeutic target for UBC management.

## AUTHOR CONTRIBUTIONS

**Chen Zhang:** Data curation (equal); formal analysis (equal); investigation (equal); resources (equal); writing – original draft (equal). **Maoyu Wang:** Data curation (equal); formal analysis (equal); investigation (equal); resources (equal); writing – original draft (equal). **Yidie Ying:** Data curation (equal); formal analysis (equal); investigation (equal); resources (equal); writing – original draft (equal). **Fang Meng:** Funding acquisition (equal); software (equal); validation (equal); visualization (equal). **Hongliang Gao:** Conceptualization (equal); software (equal); validation (equal). **Shuxiong Zeng:** Writing – review and editing (equal). **Yasheng Zhu:** Methodology (equal); writing – review and editing (equal). **Anwei Liu:** Writing – review and editing (equal). **Zhensheng Zhang:** Conceptualization (equal); methodology (equal); project administration (equal); supervision (equal). **Chuanliang Xu:** Conceptualization (equal); methodology (equal); supervision (equal).

## FUNDING INFORMATION

This research was financed by National Natural Science Foundation of China (81772720, 81972391), and the “Voyaging Talents” Fund of The PLA Naval Medical University (2021008149). The funder did not participate in the design of the study and collection, analysis, interpretation of data or in writing the manuscript.

## CONFLICT OF INTEREST STATEMENT

The authors declare that they have no competing interests.

## ETHICS STATEMENT

The research was carried out in accordance with the Declaration of Helsinki. All clinical samples obtained were approved by the ethical board of Changhai Hospital (approval #: CHEC2019‐134). Written informed consent was obtained from all patients.

## Supporting information


Table S1.

## Data Availability

All data generated or analysed during this study are included in this published article.
