# Supplementary material for: Knockdown of kinesin family member 4A inhibits cell proliferation, migration, and invasion while promoting apoptosis of urothelial bladder carcinoma cells
Source: Cancer Med. 2023 Apr 11;12(11):12581–92. doi: 10.1002/cam4.5932 (PMC10278490; doi:10.1002/cam4.5932)
Supplement: Supplementary file 1 — Table S1. [file CAM4-12-12581-s001.docx]

**Supplemental Table 1** Clinical characteristics of 10 patients with urothelial bladder carcinoma for RNA sequencing.

| Characteristics | n |
| --- | --- |
| Sex |  |
| Male | 7 |
| Female | 3 |
| Age, years |  |
| ≤ 65 | 2 |
| ＞ 65 | 8 |
| Tumor stage |  |
| Ta | 3 |
| T1 | 2 |
| T2 | 3 |
| T4 | 2 |
| Tumor grade |  |
| Low | 2 |
| High | 8 |

**Supplemental Table 2** Clinical characteristics of 16 patients with urothelial bladder carcinoma for Western blot analysis of KIF4A expression.

| Characteristics | n |
| --- | --- |
| Sex |  |
| Male | 12 |
| Female | 4 |
| Age, years |  |
| ≤ 65 | 6 |
| ＞ 65 | 10 |
| Tumor stage |  |
| Ta | 3 |
| T1 | 3 |
| T2 | 3 |
| T3 | 4 |
| T4 | 3 |
| Tumor grade |  |
| Low | 6 |
| High | 10 |
| Tumor classification |  |
| Non-muscle invasive bladder cance | 6 |
| Muscle invasive bladder cancer | 10 |
